# Supplementary figures and images for: Real-time emotion detection by quantitative facial motion analysis
Source: PLoS One. 2023 Mar 10;18(3):e0282730. doi: 10.1371/journal.pone.0282730 (PMC10004542; doi:10.1371/journal.pone.0282730)

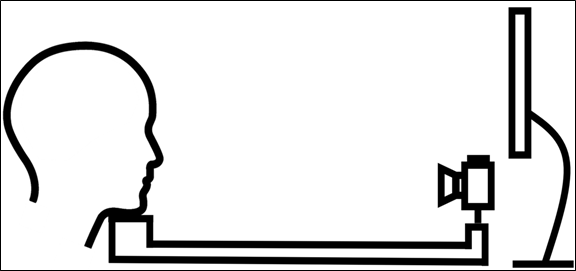

Supplement: S1 Fig — Participants placed their chin on the chinrest, which is attached to the camera mount to allow recording of the face from a fixed distance. Behind the camera is the screen that presents the images during recording. (PNG) [file pone.0282730.s001.png]

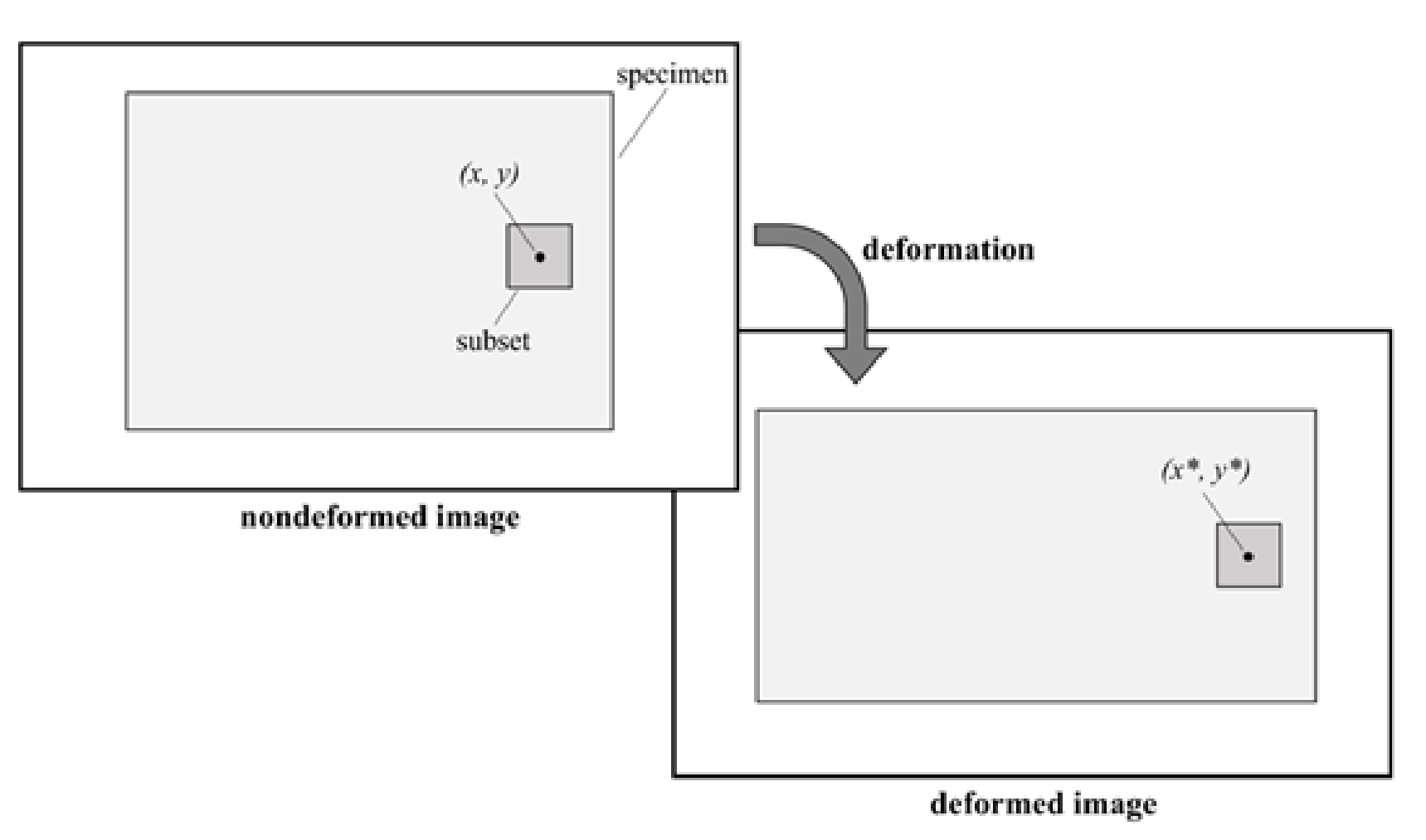

Supplement: S2 Fig — (PNG) [file pone.0282730.s002.png]
