# Supplementary material for: Real-time emotion detection by quantitative facial motion analysis
Source: PLoS One. 2023 Mar 10;18(3):e0282730. doi: 10.1371/journal.pone.0282730 (PMC10004542; doi:10.1371/journal.pone.0282730)
Supplement: S1 Table — (DOCX) [file pone.0282730.s003.docx]

**S1 Table.** **Architecture of the 3D-convolutional neural network used for the CNN emotion classifier**.

| Layer (type) | Output Shape | Number of Parameters |
| --- | --- | --- |
| Convolution 3D | (8, 30, 33, 32) | 2624 |
| Leaky ReLU | (8, 30, 33, 32) | 0 |
| Max Pooling 3D | (4, 15, 11, 32) | 0 |
| Batch Normalization | (4, 15, 11, 32) | 128 |
| Convolution 3D | (2, 13, 3, 256) | 663808 |
| Leaky ReLU | (2, 13, 3, 256) | 0 |
| Max Pooling 3D | (1, 6, 1, 256) | 0 |
| Batch Normalization | (1, 6, 1, 256) | 1024 |
| Global Average Pooling 3D | (256) | 0 |
| Dense | (256) | 65792 |
| Leaky ReLU | (256) | 0 |
| Dropout | (256) | 0 |
| Dense | (3) | 771 |
|  |  |  |
| Total parameters: | 734,147 |  |
| Trainable parameters: | 733,571 |  |
| Non-trainable parameters: | 576 |  |

Abbreviations: CNN (convolutional neural network).
